# Chemotherapy combined with immunotherapy in a patient with multiple primary gastric and rectal cancers with good prognosis: A case report

**DOI:** 10.1097/MD.0000000000040699

**Published:** 2024-11-29

**Authors:** Jibang Peng, Zhu Zhu, Min Shi, Weikang Shao, Xiang Ji, Chang Liu, Dayang Zhou, Xueqin Wang, Jian Huang

**Affiliations:** a Department of Oncology, First Affiliated Hospital of Kunming Medical University, Kunming, Yunnan Province, China; b Department of Pathology, Sichuan Clinical Research Center for Cancer, Sichuan Cancer Hospital & Institute, Sichuan Cancer Center, Affiliated Cancer Hospital of University of Electronic Science and Technology of China, Chengdu, China; c Genecast Biotechnology Co., Ltd., Wuxi, China.

**Keywords:** MSI-H, multiple primary cancers, next-generation sequencing

## Abstract

**Rationale:**

Multiple primary cancer is common in clinical practice, but its diagnosis process is complicated, and relevant genetic testing is required to assist in diagnosis when necessary. The formulation of treatment strategies for multiple primary cancer is a highly personalized process. In this article, we introduce a case of a patient with rectal cancer and gastric cancer who was diagnosed with multiple primary cancers, to investigate and explore the clinical application value of next-generation sequencing (NGS) testing in patients with multiple primary gastric and colorectal cancers.

**Patient concerns:**

A 74-year-old male patient had a mass at the anal verge.

**Diagnoses:**

Endoscopy, imaging studies, and pathological examinations showed adenocarcinoma in both the rectal and gastric antral regions. Genetic testing confirmed the diagnosis of multiple primary cancer.

**Interventions:**

The patient received 8 cycles of neoadjuvant chemotherapy combined with immunotherapy and underwent laparoscopic radical resection for rectal cancer. Postoperative adjuvant chemotherapy (XELOX) supplemented with PD-1 immunotherapy, and Camrelizumab was continued.

**Outcomes:**

Gastric lesions continued to regress and eventually disappeared completely at the end of adjuvant therapy.

**Lessons:**

According to the results of NGS testing, the multiple primary cancers’ patient received personalized treatment and ultimately achieved clinical complete remission. This case highlights the critical role of genetic testing in accurately identifying multiple primary cancer and the value of personalized guidance for patient treatment using NGS in clinical practice.

## 1. Introduction

Multiple primary cancers (MPCs) are defined as the occurrence of tumors in the same individual either simultaneously or sequentially over time. This concept was initially introduced by Billroth in 1889 when he documented a case involving the presence of multiple primary cancers, specifically a secondary gastric cancer following primary epithelial cancer of the external ear.^[1]^ MPCs in the gastrointestinal tract were once perceived as a rare condition, posing a histopathological challenge in distinguishing them from metastatic diseases.^[2]^ The importance of next-generation sequencing (NGS) in diagnosing and guiding the treatment of cancer patients has gained widespread recognition. However, there have been limited instances of its clinical utilization in differentiating multiple primary cancers. In recent years, as diagnostic techniques have advanced, NGS of lesion tissues has emerged as a new trend for auxiliary identification in cases where conventional pathological examinations fail to provide definitive answers for multiple primary cancers.^[3,4]^ A 68-year-old female patient discovered new liver lesions just 2.5 months after being diagnosed with adrenocortical carcinoma. Genome analysis revealed distinct mutation statuses for multiple genes, including AKT2 and NOTCH2, in these 2 lesions. Following a comprehensive diagnostic process, which included immunohistochemistry, the patient was ultimately diagnosed with a second primary neuroendocrine tumor.^[5]^

Moreover, NGS holds the capacity to pinpoint actionable mutations, facilitating the selection of more precise drug therapies. The identification of MPCs can, in many instances, significantly influence treatment decisions.^[6]^ For instance, ERBB2 amplification is a prevalent occurrence in gastric tumors, ranging from 2% to 27% prevalence.^[7]^ In the KEYNOTE-811 study, which focused on unresectable or metastatic HER2-positive gastric adenocarcinoma, the combination of trastuzumab with pembrolizumab alongside chemotherapy produced substantial reductions in tumor size.^[8]^ In parallel, the CLASSIC and MAGIC trials have identified microsatellite instability-high (MSI-H) status as a potential favorable prognostic factor for resectable gastric cancer (GC) within the context of neoadjuvant/adjuvant chemotherapy.^[9–11]^ Although MSI-H is relatively common in both colorectal and gastric cancers, its applicability to international neoadjuvant treatment for gastric cancer remains a subject of debate, necessitating further validation through comprehensive sample studies.^[12]^ The 2022 NCCN guidelines included 6 cases of locally advanced, nonmetastatic MSI-H GC, demonstrating improved prognosis and increased survival rates with the utilization of neoadjuvant chemotherapy in conjunction with pembrolizumab compared to chemotherapy alone.^[13]^ In order to explore the pathogenesis of multiple primary cancers in the gastrointestinal tract, Kim conducted MSI testing on patients and found that among 17 patients with colon and gastric cancer, 17.7% of them had both MSI+ in tumors of both organs. Therefore, it is believed that genetic defects in mismatch repair may be the cause of some gastrointestinal dual primary cancers.^[14]^ Through genetic testing of 10 patients with gastrointestinal double primary cancer, it was found that the APC-E-cadherin upregulation pathway and abnormal tumor suppression pathway may be activated synergistically or separately.^[15]^ At the same time, the intricate interaction between CRC and GC poses a huge challenge to surgical oncology. Heterotemporal cases with a second primary cancer detected more than 6 months after the first diagnosis have a better prognosis, emphasizing the importance of early detection and treatment.^[16]^ Nonetheless, the intricacies of treating dual primary gastrointestinal cancers surpass those of single primary cancers, and the potential advantages of neoadjuvant chemotherapy combined with immunotherapy in managing multiple primary cancers warrant additional validation.

## 2. Case presentation

**Figure 1.**
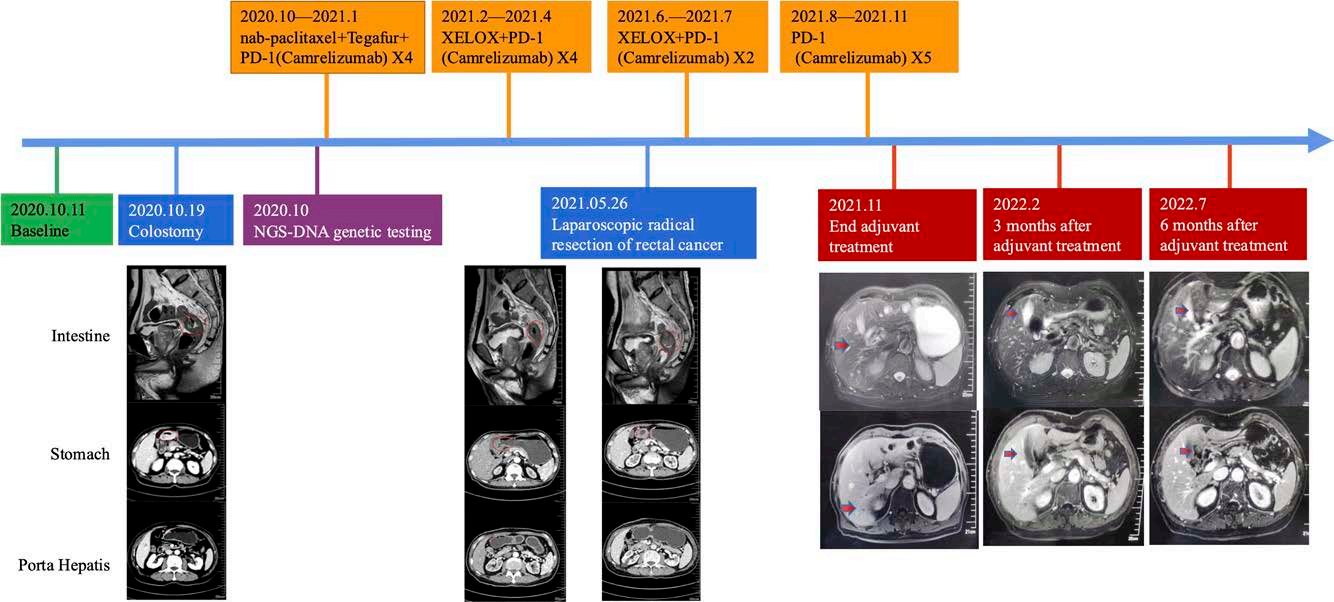
The complete treatment process of this patient includes treatment modalities and imaging examination results. The green color represents the initial diagnosis time point, the orange color represents time points for drug treatments, and the blue color represents time point for surgery, the red color represents follow-up time points, and the purple color represents the time point of NGS genetic testing. The red circles indicate tumor lesions.

In October 2020, a 74-year-old male patient presented with a challenging case involving multiple primary gastric cancer and rectal cancer when admitted to the First Affiliated Hospital of Kunming Medical University. The initial diagnostic workup included endoscopy, imaging studies, and pathological tests, which collectively revealed a tumor measuring 5.1 cm × 1.6 cm, situated 8.5 cm from the anal verge. In addition, irregular thickening of the gastric wall in the gastric antrum was observed, along with a mass in the hepatoduodenal ligament. The pathology reports indicated moderately to well-differentiated adenocarcinoma in the rectal region and moderately to poorly differentiated adenocarcinoma in the gastric antrum. Immunohistochemical results showed Syn(‐), CgA(‐), CK widespread(+), VIM(‐), CK8(+), CK18(+), KI-67(+ approximately 90%), and CD56(‐). A PET/CT scan revealed thickening of the walls in the gastric antrum, the proximal part of the duodenum, the transverse colon, and the rectosigmoid junction, with varying degrees of increased glucose metabolism, suggestive of malignancy. Additionally, there was a lesion above the pancreatic head, and small lymph nodes in the hepatopancreatic region exhibited high glucose metabolism, indicating a potential invasion of the pancreatic head. Following a comprehensive evaluation, the gastric cancer was classified as cT3N1M0, and the rectal cancer as cT4aN2M0. To ascertain precise drug treatment and the molecular subtypes of the 2 lesions, samples were sent to Genecast Biotechnology Co., Ltd., Wuxi, China, a laboratory accredited by the College of American Pathologists, CLIA, and ISO15189 for NGS-DNA analysis, encompassing 769 cancer-related genes. After thorough discussions with the patient and family members, a colostomy was performed in October 2020 to address the issue of intestinal obstruction. The patient’s treatment timeline is depicted in Figure 1.

**Figure 2.**
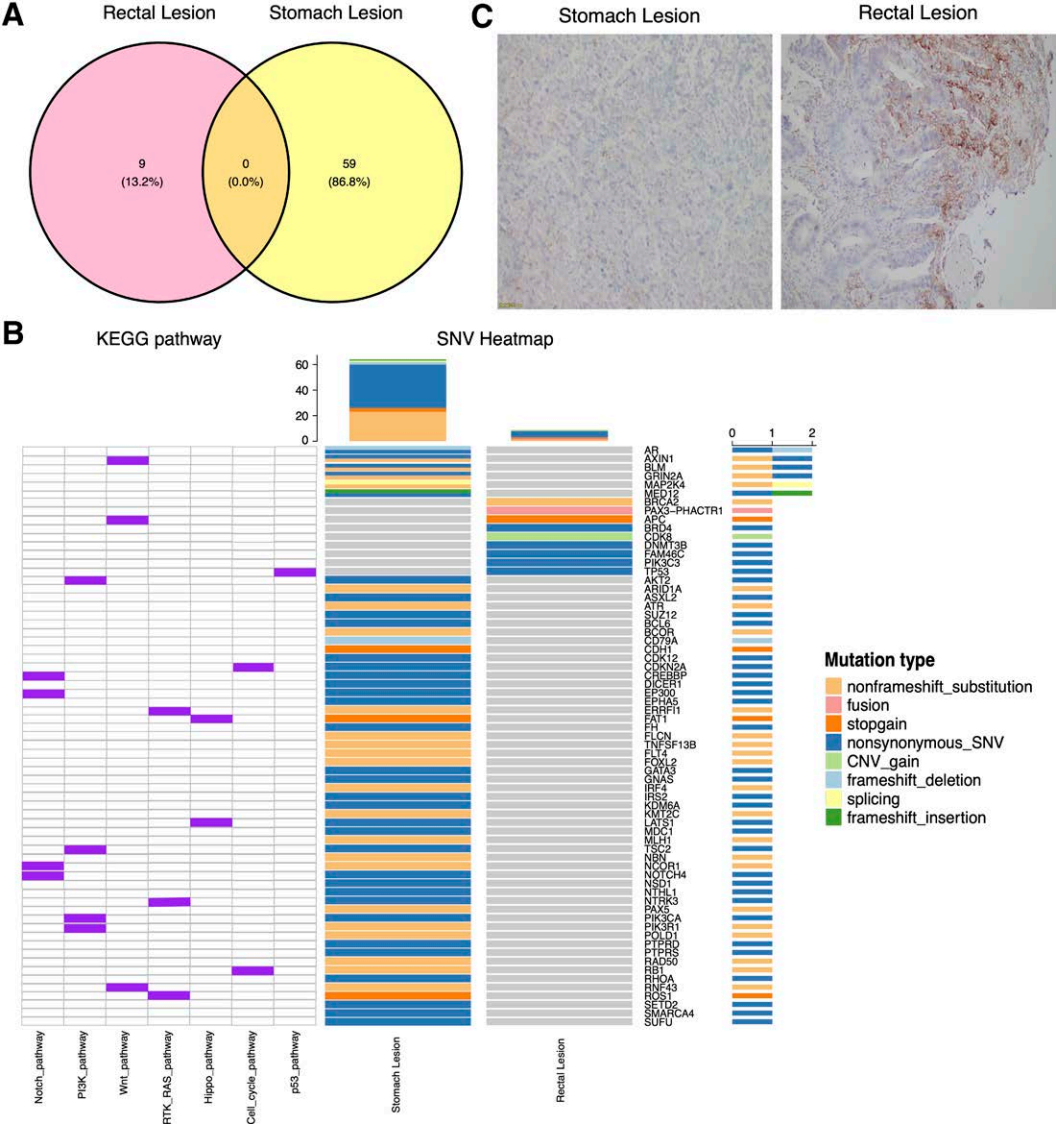
NGS and PD-L1 test results of 2 lesions in the rectal and stomach of MPCs patient. (A) Venn plot shows the number of mutated genes and the number of intersection genes in the 2 lesions. (B) Gene mutation landscape of the 2 lesions. (C) The PD-L1 (22C3) protein expression detection results of the 2 puncture tissues (formalin).

The NGS results of rectal and stomach (puncture) tumor revealed no common mutations between the 2 lesions (Table 1). The rectal exhibited 9 mutations, while the stomach displayed 59 mutations, with no overlapping mutated genes between the 2 tissues (Fig. 2A). The gastric lesion demonstrated a wild-type ERBB2 (Fig. 2B and Table 1), high tumor mutation burden (TMB-H, 35.67 mutation/Mb), MSI-H, and PD-L1 negativity (tumor cell proportion score = 0%, combined positive score < 1, Fig. 2C). In contrast, the rectal lesion exhibited a wild-type KRAS (Fig. 2B and Table 1), intermediate TMB (3.84 mutation/Mb), microsatellite stability (MSS), PD-L1 positivity (TPS = 10%, CPS = 11, Fig. 2C), and a BRCA2 mutation (p. N986Kfs*2, 3.88%) (Fig. 2B and Table 1). Given the PD-L1 positivity in the rectal lesion and high tumor mutation burden and MSI-H in the gastric lesion, after comprehensive discussions with the patient, it was decided to supplement the chemotherapy regimen with immunotherapy. From late October 2020 to January 2021, the patient received 4 cycles of nab-paclitaxel and tegafur combined with PD-1 (Camrelizumab). To the surprise of the medical team, the intestinal lesion exhibited stable disease, while the hepatic hilar nodule and gastric mass decreased in size. To further reduce the size of the intestinal lesion, the patient transitioned to the XELOX regimen (Oxaliplatin and Capecitabine) in combination with PD-1 immunotherapy (Camrelizumab) for 4 cycles. By May 2021, the patient had completed the full course of neoadjuvant chemotherapy combined with immunotherapy. The patient tolerated the treatment well. The intestinal lesion remained stable, while the hepatic hilar nodule and gastric lesion exhibited a continuous partial response and ultimately achieved disappearance. Imaging assessments were employed to evaluate the surgical opportunity for the bowel lesion. Based on the imaging examination results, laparoscopic radical resection of rectal cancer was performed on May 26, 2021 (Fig. 1), subsequently downgrading the bowel cancer to cT2N0M0.

**Table 1:**
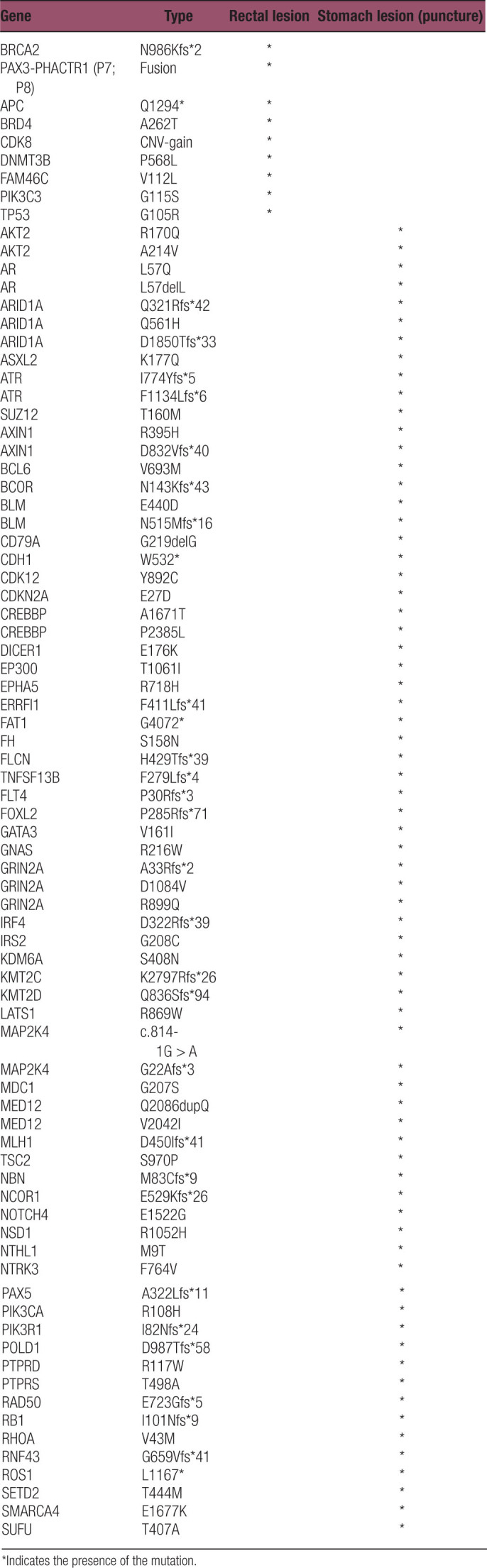
Results of next-generation sequencing for gastric and rectal cancer lesions in patient.

To consolidate the treatment effect, adjuvant chemotherapy combined with immunotherapy was initiated in June 2021. The patient underwent 2 cycles of the XELOX regimen (oxaliplatin + capecitabine) combined with PD-1 immunotherapy (Camrelizumab), followed by 5 cycles of PD-1 immunotherapy alone (Camrelizumab). In November 2021, gastroscopy demonstrated the disappearance of ulcerative lesions, and the gastric lesion achieved clinical complete remission, downgrading to cT0N0M0. The gastric lesion, previously a moderately to poorly differentiated adenocarcinoma, no longer exhibited significant thickening of the gastric wall and displayed no significant increase in glucose metabolism. Notably, the nodules in the hepatic hilar region and the gap between the pancreatic head and liver had significantly decreased in size, with a noticeable reduction in glucose metabolism compared to previous scans (Fig. 1). PET/CT scans indicated no definite density shadows or areas of increased glucose metabolism in the lower segment of the rectum.

The patient continued to receive regular follow-up examinations every 3 months. Magnetic resonance imaging conducted in November 2021 and February 2022 revealed similar abnormal signals in the hepatic hilar region and above the pancreatic head as before. As of June 2022, the patient remained alive and in good physical condition, with the MRI showing no definite display of abnormal signal lesions in the hepatic hilar region and above the pancreatic head (Fig. 1). There were no signs of tumor recurrence in the stomach and rectum.

## 3. Discussion and conclusions

The incidence of MPCs has been steadily increasing over the years.^[2]^ In this particular case, the initial diagnosis indicated the presence of MPCs in the form of gastric cancer and rectal cancer. Specifically, the gastric lesion was classified as moderately to poorly differentiated adenocarcinoma (cT3N1M0, exhibiting a high TMB and MSI-H), while the rectal lesion was categorized as moderately to well-differentiated adenocarcinoma (cT4aN2M0, displaying intermediate TMB and MSS).

NGS has been extensively employed in identifying MPCs across various cancer types. For instance, it has played a crucial role in distinguishing primary lung tumors from lung recurrent and metastatic tumors.^[17,18]^ One patient had 2 tumors located in the right upper lobe of the lung and the left rib. The right upper lobe was pathologically diagnosed as non-small cell lung cancer, and the left rib was diagnosed as a second primary cancer by morphology and immunohistochemistry. Genomic testing of the 2 tissues revealed a significant overlap in the genomic signatures of the 2 tumors, both harboring the oncogenic KRAS p.Q61L mutation. The final diagnosis was revised from the second primary cancer to recurrent metastatic NSCLC.^[5,19]^ In addition, NGS also assisted in the identification of 4 primary tumor cases of bone tumor, malignant bladder tumor, malignant melanoma, and intrahepatic cholangiocarcinoma, as well as 8 MPCs such as endometrial adenocarcinoma and renal cell carcinoma.^[6,20,21]^ In 2022, Chiara Romano et al reported a case of colorectal cancer found in a man 2 years after the diagnosis of poorly differentiated gastric adenocarcinoma. The second tumor was initially considered to be a recurrence of gastric cancer. In fact, through NGS detection of cancer-related genes, only somatic mutations were found in the first gastric cancer, and germline mutations in 3 genes, PDGFRA, APC, and TP53. The patient was finally diagnosed as a patient with multiple primary gastrointestinal diseases by NGS.^[3]^ Although the use of NGS for identifying double primary gastrointestinal cancers has been demonstrated in previous studies, it has not yet been universally recommended in guidelines or reached expert consensus. Nevertheless, this case provides further support for the utility of NGS in clinical practice.

In our case study, NGS was also used to better elucidate the relationship between multiple lesions and explore more effective treatment options. Despite both lesions being confirmed as adenocarcinoma through molecular pathological examination, NGS analysis revealed no common mutations between the 2 lesions. Additionally, the TMB values and MSI status differed, confirming that the gastric and colorectal lesions represented independent primary tumors. This provides evidence for the utility of NGS in facilitating the diagnosis and treatment decision-making for MPCs of gastric and colorectal cancers, supporting the necessity of genetic testing in the diagnosis of multiple primary cancers. In future clinical practice, genetic testing for each MPC may be necessary to confirm the diagnosis, identify potential molecular features, and guide treatment choices.

NGS not only assists in differentiating MPCs but also guides personalized treatment for this complex condition. Approximately 5% of gastric cancer patients exhibit MSI-H status.^[22]^ In this case, the gastric lesion is ERBB2 wild-type and MSI-H, making it unsuitable for targeted therapy with ERBB2 inhibitors. However, numerous domestic and international studies have demonstrated the safety and efficacy of neoadjuvant chemotherapy for advanced gastric cancer.^[23,24]^ The 2022 NCCN guidelines highlight the importance of exploring the prognosis and treatment options for MSI-H gastric adenocarcinoma in the perioperative period. Neoadjuvant therapy is recommended as a level II treatment modality, with a focus on perioperative immunotherapy. For patients with PD-L1 CPS < 5 or those with inaccessible testing results, the first-line treatment now includes the addition of FOLFOX/XELOX combined with ramucirumab, which is a level II recommendation. Recent small-scale studies have shown that neoadjuvant chemotherapy combined with immunotherapy can benefit ERBB2-negative gastric cancer. The results of the phase III RCT Checkmate 649 study, particularly in the MSI-H subgroup, further support the effectiveness of combining nivolumab and ipilimumab or nivolumab with chemotherapy over chemotherapy alone.^[25,26]^ However, the efficacy of immunotherapy in patients with multiple primary gastric and rectal cancers is unclear. In this case, neoadjuvant immunotherapy combined with chemotherapy was attempted for the MSI-H gastric cancer patient, resulting in significant remission of the gastric tumor, consistent with findings in other reported cases.

Although there is a chance for curative resection of rectal cancer, effective tumor reduction through neoadjuvant therapy poses a significant challenge to overall treatment. Traditionally, MSS colorectal cancer patients typically exhibit low-frequency somatic mutations, low TMB, fewer neoantigens, lower levels of CD8+ tumor-infiltrating lymphocytes, and an immunosuppressive tumor microenvironment, leading to poor response to immunotherapy.^[27]^ Immunotherapy for these patients requires approaches such as increasing tumor antigen presentation and enhancing the recognition of tumor antigens by effector cells. Neoadjuvant therapy combined with chemoradiotherapy can improve the tumor microenvironment and increase sensitivity to chemotherapy, showing promising initial data. However, due to the limitation of the number of tissue samples in this case, only NGS testing can be performed, and multiplex immunohistochemical or other tests cannot be performed. Therefore, it is not possible to explore the immune microenvironment of multiple primary gastrointestinal cancers, and to explore the molecular mechanisms behind the positive response of such patients to treatment.

The comprehensive treatment strategy in this case was to control the double lesion with neoadjuvant chemotherapy combined with immunotherapy, and elective radical resection of the rectal cancer followed by adjuvant immunotherapy to prolong the patient’s overall survival as much as possible. Genetic testing played a crucial role in guiding treatment selection, despite some biomarkers exhibiting conflicting results between the patient’s 2 lesions, such as MSS type and high PD-L1 expression in the bowel cancer lesions. Tailoring treatment regimens to address both gastric and rectal cancers involved selecting drugs that covered both tumor types and choosing similar drugs with the same mechanism of action. After completing 8 cycles of neoadjuvant therapy, the patient achieved sustained stable disease for the bowel cancer and a partial response for the gastric cancer. Following adjuvant therapy, the gastric cancer lesions completely disappeared, achieving clinical complete remission. These findings not only provided a personalized treatment plan for the patient but also offered important data support for future research and treatment strategies of multiple primary cancers. Due to the rarity of treating multiple patients with primary gastric cancer and colorectal cancer in clinical practice, this CASE study has certain limitations and may only present partial population’s condition. The guiding value of NGS in the treatment of multiple primary gastric cancer and colorectal cancer patients requires more sample size for research. We will continue to follow up with patients to observe the long-term survival.

## Author contributions

**Data curation:** Jibang Peng.

**Formal analysis:** Zhu Zhu.

**Funding acquisition:** Zhu Zhu, Min Shi.

**Investigation:** Min Shi, Xiang Ji, Chang Liu, Dayang Zhou.

**Methodology:** Weikang Shao.

**Project administration:** Jibang Peng, Min Shi, Jian Huang.

**Resources:** Xiang Ji, Chang Liu, Dayang Zhou.

**Supervision:** Zhu Zhu, Jian Huang.

**Visualization:** Weikang Shao.

**Writing – original draft:** Jibang Peng, Weikang Shao.

**Writing – review & editing:** xueqin Wang, Jian Huang.

## References

[1] Billroth T. Die Allgemeine Chirurgische Pathologie und Therapie. G. Reimer; 1889.

[2] Li K Gong J Zheng Q. Preliminary study on the molecular features of mutation in multiple primary oral cancer by whole exome sequencing. Front Oncol. 2022;12:971546. 36338765 10.3389/fonc.2022.971546 PMC9632273

[3] Chiara R Sandra DG Stella PM. Multiple primary malignances managed with surgical excision: a case report with next generation sequencing analysis. Mol Biol Rep. 2022;49:9059–64. 35715605 10.1007/s11033-022-07630-8

[4] Simona S Simona DS Brunella P. Next-generation sequencing: advances and applications in cancer diagnosis. Oncotargets Ther. 2016;9:7355–65. 10.2147/OTT.S99807 PMC5144906 27980425

[5] Weinberg B Lee K Ou TK. Comprehensive genomic profiling aids in distinguishing metastatic recurrence from second primary cancers. Oncologist. 2017;22. 10.1634/theoncologist.2015-0511 PMC5330711 28193735

[6] Ma X-Y Tian K Sun P-F. Multiple primary malignant neoplasm: case report and comprehensive literature review. Front Oncol. 2023;12:1090634. 36686734 10.3389/fonc.2022.1090634 PMC9846320

[7] Forbes SA David B Prasad G. COSMIC: exploring the world’s knowledge of somatic mutations in human cancer. Nucleic Acids Res. 2015;43:805–11. 10.1093/nar/gku1075 PMC4383913 25355519

[8] Janjigian YY Kawazoe A Yaez P. The KEYNOTE-811 trial of dual PD-1 and HER2 blockade in HER2-positive gastric cancer. Nature. 2021;600:727–30. 34912120 10.1038/s41586-021-04161-3 PMC8959470

[9] Noh SH Park SR Yang HK. Adjuvant capecitabine plus oxaliplatin for gastric cancer after D2 gastrectomy (CLASSIC): 5-year follow-up of an open-label, randomised phase 3 trial. Lancet Oncol. 2014;15:1389–96. 25439693 10.1016/S1470-2045(14)70473-5

[10] Samalin E Ychou M. Neoadjuvant therapy for gastroesophageal adenocarcinoma. World J Clin Onco. 2016;7:284. 10.5306/wjco.v7.i3.284 PMC4896896 27298768

[11] Smyth EC Wotherspoon A Peckitt C Gonzalez D Cunningham D. Mismatch repair deficiency, microsatellite instability, and survival: an exploratory analysis of the Medical Research Council Adjuvant Gastric Infusional Chemotherapy (MAGIC) Trial. Jama Oncology. 2017;3:1197–203. 28241187 10.1001/jamaoncol.2016.6762 PMC5824280

[12] Pietrantonio F Miceli R Raimondi A Kim YW Smyth EC. Individual patient data meta-analysis of the value of microsatellite instability as a biomarker in gastric cancer. J Clin Oncol. 2019;37:3392–400. 31513484 10.1200/JCO.19.01124

[13] Liu L Woo Y D’Apuzzo M. Immunotherapy-based neoadjuvant treatment of advanced microsatellite instability—high gastric cancer: a case series. J Natl Compr Canc Netw. 2022;20:857–65. 35948034 10.6004/jnccn.2022.7023

[14] Kim HS Cho NB Yoo JH. Microsatellite instability in double primary cancers of the colorectum and stomach. Modern Pathol. 2001;14:543–8. 10.1038/modpathol.3880347 11406654

[15] Kim JC Koo KH Kim HC Kim JS Kang GH. Geno- and pheno-typic characterization in ten patients with double-primary gastric and colorectal adenocarcinomas. Int J Colorectal. 2004;19:561–8. 10.1007/s00384-004-0591-7 15083323

[16] Marano L. Dual primary gastric and colorectal cancer: a complex challenge in surgical oncology. World J Gastrointest Oncol. 2023;15:2049–52. 38173432 10.4251/wjgo.v15.i12.2049 PMC10758648

[17] Zhao L Liu C Xie G Wu F Hu C. Multiple primary lung cancers: a new challenge in the era of precision medicine. Cancer Manag Res. 2020;12:10361–74. 33116891 10.2147/CMAR.S268081 PMC7585808

[18] Ezer N Wang H Corredor AG. Integrating NGS-derived mutational profiling in the diagnosis of multiple lung adenocarcinomas. Cancer Treat Res Commun. 2021;29:100484. 34773797 10.1016/j.ctarc.2021.100484

[19] Frampton GM Fichtenholtz A Otto GA. Development and validation of a clinical cancer genomic profiling test based on massively parallel DNA sequencing. Nat Biotechnol. 2013;31:1023–31. 24142049 10.1038/nbt.2696 PMC5710001

[20] Vogt A Schmid S Heinimann K. Multiple primary tumours: challenges and approaches, a review. Esmo Open. 2017;2:e000172. 28761745 10.1136/esmoopen-2017-000172 PMC5519797

[21] Slem A Abu-Hijlih R Abdelrahman F. Eight primary malignancies: case report and review of literature. Hematol Oncol Stem Cell Ther. 2011;4:185–7. 22198190 10.5144/1658-3876.2011.185

[22] Le DT Durham JN Smith KN. Mismatch repair deficiency predicts response of solid tumors to PD-1 blockade. Science. 2017;357:409–13. 28596308 10.1126/science.aan6733 PMC5576142

[23] Song Z Wu Y Yang J Yang D Fang X. Progress in the treatment of advanced gastric cancer. Tumour Biol. 2017;39:1010428317714626. 28671042 10.1177/1010428317714626

[24] Li ZY Koh CE Bu ZD. Neoadjuvant chemotherapy with FOLFOX: improved outcomes in Chinese patients with locally advanced gastric cancer. J Surg Oncol. 2012;105:793–9. 22189752 10.1002/jso.23009

[25] Janjigian YY Shitara K Moehler M. First-line nivolumab plus chemotherapy versus chemotherapy alone for advanced gastric, gastro-oesophageal junction, and oesophageal adenocarcinoma (CheckMate 649): a randomised, open-label, phase 3 trial. Lancet. 2021;398:27–40. 34102137 10.1016/S0140-6736(21)00797-2 PMC8436782

[26] Takei S Kawazoe A Shitara K. The new era of immunotherapy in gastric cancer. Cancers. 2022;14:1054. 35205802 10.3390/cancers14041054 PMC8870470

[27] Liu S Zhang Y Lin Y Wang P Pan Y. Case report: The MSI-L/p-MMR metastatic rectal cancer patient who failed systemic therapy responds to anti-PD-1 immunotherapy after stereotactic body radiation-therapy. Front Immunol. 2022;13:981527. 36119063 10.3389/fimmu.2022.981527 PMC9479073

